# A Systematic Review on Whether Retinal Artery Microaneurysms Are Reliable Predictors of Coronary Artery Disease Severity

**DOI:** 10.7759/cureus.92813

**Published:** 2025-09-20

**Authors:** Ameer A Khan, Muhammad Saleem, Munir Khan, Majd El Rifai, Thet Myat Noe, OIiver Biggs, Muhmood Elnaiem, Faqir Mohammad Khan Karim

**Affiliations:** 1 Cardiology, Tameside General Hospital, Ashton-Under-Lyne, GBR; 2 Cardiology, Faculty of Medicine, University of Leeds, Leeds, GBR; 3 Medicine and Surgery, St James's University Hospital, Leeds, GBR; 4 General Medicine, Leeds Teaching Hospital, Leeds , GBR; 5 Medicine, St James's University Hospital, Leeds, GBR; 6 Medicine, University of Leeds, Leeds, GBR

**Keywords:** cad: coronary artery disease, predictors of cad, preventive medicine, retina, retinal artery microaneurysms

## Abstract

Coronary artery disease (CAD) remains one of the leading causes of mortality in the UK. The growing and ageing population has led to increasing CAD prevalence and escalating healthcare costs. Current diagnostic tools like coronary angiography are too invasive for large population-level screening. Retinal artery microaneurysms (RAMs) are caused by microvascular damage and are detectable via non-invasive eye screening. This raises the possibility of a novel screening tool to help identify individuals at risk of CAD. This systematic review investigates whether RAMs are a reliable predictor of CAD severity. A Preferred Reporting Items for Systematic Review and Meta-Analysis (PRISMA)-compliant approach was used in the review, and eligible studies were selected. Preliminary evidence suggests that RAMs may serve as a non-invasive indicator of CAD severity, enabling more non-invasive approaches in helping cardiovascular risk stratification of the general population. Integration of retinal imaging into cardiovascular risk assessment protocols could hold promise for the future of preventative cardiology.

## Introduction and background

Background

Coronary artery disease (CAD) is an umbrella term encompassing varying degrees of cardiac ischaemia caused by atherosclerosis in the coronary arteries. The buildup of atherosclerotic plaque leads to progressive luminal narrowing of the coronary arteries, which can eventually compromise cardiac cell oxygenation. Retinal artery microaneurysms (RAMs) are small, focal dilatations of retinal capillaries resulting from structural weakening of the capillary wall. Basement membrane thickening due to chronic systemic insult leads to local hypoxia, which in turn causes loss of the supporting pericytes [1]. Chronic hyperglycaemia is the most common cause of this sequence, and hypertension is also a recognised risk factor; albeit, in hypertensive retinopathy, microaneurysms typically appear in the latter stages, where vascular changes are already established [2]. Retinal microaneurysms are the first observable manifestation of diabetic retinopathy, staging from mild non-proliferative to severe proliferative retinopathy, and play a crucial role in the established clinical pathways for screening of diabetic eye disease [3,4].

Rationale

The progress made in the management of acute coronary syndromes over the last half century represents one of the most meaningful achievements in modern medicine. In the 1960s, around the time the first coronary artery bypass graft was performed in the UK, upwards of 70% of heart attacks were fatal. Today, this trend has reversed, with over 70% of patients surviving the acute ischaemic event [5]. This dramatic improvement creates a new challenge for the NHS and other healthcare systems serving aging populations. As the UK population continues to increase in both size and age, the prevalence of CAD rises, driven not by increasing incidence, but by improving survival [5,6]. This generates a growing burden in managing both the physical sequelae (e.g., heart failure) and social consequences (e.g., reduced ability to carry out daily activities). Financially, the cost of cardiovascular disease to the UK economy has risen from £15 billion in 2014 to £28 billion in recent estimates, an 86.7% increase in a remarkably short time [5]. On top of this economic impact, the cost in human life remains staggeringly high: cardiovascular disease accounts for up to 25% of UK deaths according to the British Heart Foundation 2025 statistics [5]. Whilst patients may enter cardiovascular monitoring through biochemical abnormalities such as hypercholesterolaemia, blood markers alone do not inform us about anatomical narrowing of the coronary vessels. In order to achieve this, coronary angiography must be used, a highly invasive investigation unsuitable for screening or regular surveillance. The reality is that CAD is often not investigated until patients develop symptoms of chest pain, at which point the opportunity for prevention has passed. Tools such as the QRISK calculator do offer some risk stratification, but the benefit of identifying more direct, non-invasive indicators of coronary artery status remains compelling. Retinal artery microaneurysms, already part of routine eye screening protocols, may offer a viable signal in this context [4].

Research question

Are retinal artery microaneurysms an effective predictor of CAD severity?

Objectives

The purpose of this systematic review is to assess existing research on the potential effectiveness of RAMs in estimating CAD. This will require an assessment of the degree of association between both conditions. If a predictable association does exist, this review will aim to appraise how RAMs can be used to forecast the severity of CAD and whether there are practical applications in using retinal signs as indicators in real-world clinical practice-for example, in diabetic patients undergoing eye screening or more broadly. Only articles published within the last 10 years were included, with the goal of examining the link between RAMs and CAD severity. Overall, this paper seeks to synthesise current evidence on this relationship.

## Review

Methods

In order to conduct this review, a robust search was performed to find the most relevant papers pertaining to the subject. An electronic literature search was conducted using the databases MEDLINE, PubMed and SCOPUS on the 14th of April 2025. The search terms used were “retina*” AND “arter*” AND “microaneurysm” AND (“CAD” or “coronary artery disease”). Boolean operators were utilised to help conduct a thorough search of the literature available. The search terms were used as keywords and as MeSH terms to facilitate a broad-based search. Truncation was also implemented to access a wider range of papers for the search. Initially during the literature search, all fields were included when searching the databases. This was done to help capture as many initial relevant articles as possible. Duplicate findings across databases were removed and papers were also excluded if they were not written in the English language prior to screening.

Following this, the search was narrowed to only include the search terms within titles, abstracts and keywords for inclusion. The reason for this was to ensure more precise and directly relevant results were generated in relation to the research question. Papers were also excluded if they were review papers, were not related to CAD, were book chapters, or not accessible in full. A restriction on the date of publication was imposed as being within the last 25 years. This ensured the inclusion of contemporary evidence detailing up-to-date clinical practice, diagnostic techniques and allowed accurate conclusions to be drawn.

**Figure 1:**
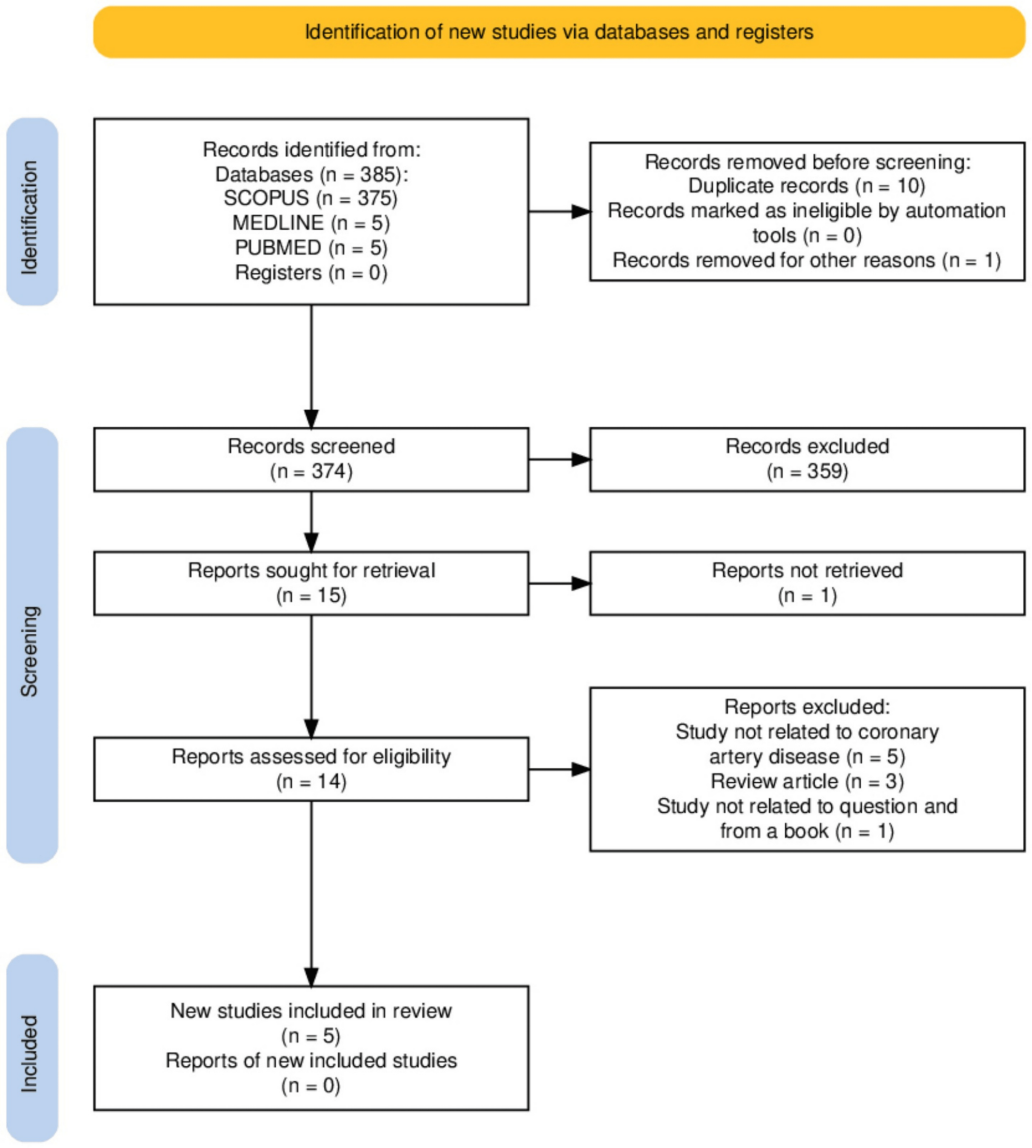
PRISMA flowchart PRISMA: Preferred Reporting Items for Systematic Review and Meta-Analysis

All relevant articles were scanned by three authors. All papers chosen to be included in the review underwent a final consensus between the three authors followed by a final re-evaluation of the chosen literature. This systematic review was completed per Preferred Reporting Items for Systematic Review and Meta-Analysis (PRISMA) [7]. The PRISMA flowchart in Figure 1 summarises our search.

A risk of bias assessment was conducted on the selected studies and is shown in Table 1. 

**Table 1:** Risk of bias assessment for selected studies CAD: Coronary Artery Disease; CHD: Coronary Heart Disease; ACS: Acute Coronary Syndromes; DR: Diabetic Retinopathy; OCT-A: Optical Coherence Tomography Angiography; FAZ: Foveal Avascular Zone

Paper	Confounding	Selection of Participants	Classification of Exposure/Intervention	Deviations from Intended Interventions	Missing Data	Measurement of Outcomes	Selection of Reported Result	Overall Risk
Wong et al. (2003) [8]	Moderate – adjusted for some vascular factors but residual confounding possible	Low – population-based elderly cohort	Low – standardised fundus photography	Low – not intervention-based	Moderate – missingness not fully detailed	Moderate – grading variability possible	Low – results broadly reported	Moderate
Campos et al. (2024) [9]	Moderate – CAD and DR both linked to multiple unadjusted lifestyle factors	Moderate – recruited from cardiology clinics; potential selection bias	Low – OCT-A & FAZ metrics well-defined	Low – no intervention deviations	Low – recent study, good data handling	Low – standardized imaging	Low – outcomes consistently reported	Moderate
Kralev et al. (2010) [10]	High – unclear adjustment for comorbidities (e.g., diabetes, hypertension)	Moderate – hospital-based ACS vs. stable CAD groups	Moderate – exposure classification partly clinical	Low – not applicable	Moderate – small sample, loss to follow-up not detailed	Moderate – subjective interpretation of retinal findings	Low – key findings reported	Serious
Duncan et al. (2002) [11]	Low – good adjustment for risk factors in a prospective cohort	Low – clear inclusion of high-risk hypertensive men	Low – standardized retinal grading	Low – no deviations	Low – prospective follow-up with good data retention	Low – outcomes measured rigorously	Low – prespecified CHD outcomes	Low
Aschauer et al. (2021) [12]	Moderate – residual confounding possible	Moderate – only CHD patients included, limits external validity	Low – advanced OCT-A/adaptive optics used	Low – not applicable	Low – data completeness adequate	Low – high-resolution imaging reduces bias	Low – multiple outcomes reported	Moderate

Results

The literature search yielded five articles suitable to be included in the review and these have been tabulated in Table 2.

**Table 2:** Results of systematic literature search CAD: Coronary Artery Disease; CHD: Coronary Heart Disease; OCT-A: Optical Coherence Tomography Angiography; FAZ: Foveal Avascular Zone; MVD: Mean Vascular Density

Paper Title	Authors (Year)	Study Population	Main Findings	Comparative Insights
The Prevalence and Risk Factors of Retinal Microvascular Abnormalities in Older Persons	Wong et al. (2003) [8]	Elderly, non-diabetic persons	Retinopathy prevalence: 8.3–9.6%. Strong association with hypertension and markers of atherosclerosis. Suggests a link to vascular damage from hypertension.	Highlights the role of hypertension-induced microvascular damage.
Association between Obstructive Coronary Disease and Diabetic Retinopathy	Campos et al. (2024) [9]	Diabetic patients with and without CAD	Diabetic retinopathy more prevalent in CAD patients. Lower MVD and FAZ circularity. CAD, diabetes duration, and insulin use independently associated with retinopathy.	Links macrovascular disease (CAD) with microvascular changes in the retina.
Microvascular Retinal Changes in Patients Presenting with Acute Coronary Syndromes	Kralev et al. (2010) [10]	Patients presenting with acute coronary syndromes vs. stable CAD	ACS patients showed a higher incidence of microaneurysms and dot haemorrhages. Retinal findings may predict acute coronary events.	Acute retinal changes can signal acute coronary syndromes.
Hypertensive Retinopathy and Incident Coronary Heart Disease in High Risk Men	Duncan et al. (2002) [11]	Hypertensive men at high risk of CHD	Hypertensive retinopathy (arteriolar narrowing) predicted 2–3x higher CHD risk, independent of other risk factors.	Emphasizes the predictive value of retinal examination for CHD risk stratification.
Identification of Subclinical Microvascular Biomarkers in Coronary Heart Disease in Retinal Imaging	Aschauer et al (2021) [12]	Patients with coronary heart disease	OCT-A and adaptive optics identified subclinical features (arteriovenous nicking, arterial narrowing, increased wall-to-lumen ratio) more common in CHD patients.	Provides imaging evidence for subclinical retinal biomarkers in CHD detection.

Discussion

The potential for retinal artery aneurysms and other retinal microvascular changes to serve as predictive markers for the severity of CAD has been a growing topic of interest. This systemic review aims to identify whether retinal artery aneurysms can effectively act as indicators for the severity of CAD. Several studies included below suggest a meaningful association between retinal microvascular abnormalities, particularly microaneurysms, and the severity of CAD.

In a comprehensive study involving over 2000 participants, Wong et al. demonstrated that retinopathy (including microaneurysms and haemorrhages) was significantly associated with the prevalence of coronary heart disease, myocardial infarction and stroke [8]. The study design focused on elderly, non-diabetic individuals who underwent retinal photography and grading of retinal microvascular characteristics [8]. The study found that retinal microvascular abnormalities are more common within older populations and associated with cardiovascular risk factors such as hypertension [8]. However, the study noted that those patients whose retinal abnormalities included retinopathy had a higher prevalence of atherosclerotic disease, even after adjusting for risk factors such as age, gender, race and blood pressure. This study demonstrates a link between MARs and CAD and therefore the need for further examination of retinal microvasculature to predict systemic vascular health and potential cardiovascular risk.

A cross-sectional study by Compos et al. investigated the association between obstructive coronary disease and diabetic retinopathy using multimodal retinal imaging techniques. They observed a significantly higher prevalence of microaneurysms in patients with CAD compared to those without (25.3 % vs 13.1 %, p = 0.043) [9]. Furthermore, ultra-widefield imaging showed that microaneurysms are more frequent in individuals with CAD than those without (34.5% vs 17.9%, p = 0.014) [9]. These findings suggest a significant association between microvascular retinal changes and CAD which reinforces the idea that retinal changes can reflect systemic vascular disease. The evidence from this study suggests that retinal imaging could be a non-invasive investigation for assessing the severity of CAD, particularly for diabetic patients and potentially in the wider population. Given the recent advancements of artificial intelligence, there may be future scope for incorporating this to the aid the screening process.

Kralev et al. assessed retinopathy changes in patients presenting with confirmed acute coronary syndromes in comparison to those with stable CAD [10]. Nineteen patients with stable CAD, and 43 patients with ACS were assessed for retinopathy changes using standardized protocols including retinal fundus photography 48 hours post-coronary angiography [10]. This study found that the patients with ACS have a higher chance for retinal microaneurysms compared to those with stable CAD (40% vs 5% OR 11.77; 95%CI 1.43-96.59; p=0.006) [10]. This striking difference suggests that the presence of retinal microaneurysms can reflect not only the presence of CAD but also indicates the likelihood of severe coronary artery narrowing. Therefore, the study by Kralev et al. provides the strongest direct link between RAMs and the severity of CAD.

In terms of predictive value, Duncan et al. showed that hypertensive retinopathy, which includes features such as microaneurysms, was independently associated with the future development of coronary heart disease in a high-risk male population [11]. This outlines the critical role retinal assessments could play in clinical evaluation and risk stratification for CAD.

Aschauer et al. stated that in populations at risk of cardiovascular conditions, retinal investigations including high-resolution quantitative and qualitative microvascular phenotyping may be used to identify subclinical CAD [12]. In 45 patients, 27 of whom have confirmed CAD, it identified microaneurysms as one of the most prevalent retinal pathologies, along with arteriovenous nicking and focal arterial narrowing [12]. This reinforces the idea that microaneurysms are a common finding in individuals with CAD and may be present even before cardiovascular symptoms manifest themselves. In addition, this study highlights the way in which non-invasive techniques such as optical coherence tomography angiography of the retina can be beneficial in helping evaluate microvascular integrity and potentially providing a predictive link between retinal findings and CAD [12].

Collectively, these studies support the hypothesis that retinal microaneurysms as part of microvascular changes may serve as a non-invasive predictive marker for CAD. The consistent association across multiple populations including patients with ACS, high risk populations, male gender and asymptomatic individuals indicates that retinal imaging can serve as an attractive tool for screening and risk stratification, potentially allowing for earlier identification of individuals at higher risk for severe CAD.

Limitations

Despite these promising findings, the studies still display limitations. Most studies were cross-sectional and therefore cannot confirm causality or predictive validity over time. Longitudinal studies (for example, that by Duncan et al. [11]) are needed to firmly establish the predictive power of retinal microaneurysms for future CAD, its severity, and any manifestation in acute coronary syndrome. Furthermore, variability in imaging modalities, diagnostic definitions, and grading systems may also negatively influence the consistency of findings across different studies. While retinal microaneurysms are associated with CAD, they are also characteristic of other systemic conditions such as diabetes and hypertension, which may act as confounding factors and obscure the extent to which microaneurysms independently predict CAD. Furthermore, some studies have relatively small sample sizes (e.g., Auscheur et al., n=45. Kralev et al., n=43), which reduces the reliability of the studies and necessitates their validation in larger cohorts. Finally, the precise pathophysiological link between retinal microvascular changes and heart disease remains unclear, highlighting the need for further research to fully establish the science behind this association. It is worth noting that the current evidence base around the presence or absence of microaneurysms does not influence the standard protocol for investigating CAD.

## Conclusions

In conclusion, retinal microaneurysms appear to be promising indicators of CAD severity and progression. Non-invasive retinal imaging presents as a valuable opportunity for early cardiovascular risk identification, particularly in asymptomatic or high-risk individuals. Retinal imaging is already a part of NHS diabetic eye screening; in the future, this could serve a dual purpose in identifying patients at risk of cardiovascular disease and allow for preventative intervention in this high-risk group. An added advantage of this from a public health perspective is that this non-invasive screening for CAD could build on already existing infrastructure in the NHS. In addition, the wider roll-out of retinal imaging in non-diabetic groups may also identify asymptomatic CAD and reduce the reliance on expensive and invasive tests such as coronary angiography. Further research will be needed to determine the most efficient screening frequency for these individuals. 

Whilst the current evidence base is limited by multiple factors including small sample sizes, cross-sectional study design and confounding variables such as diabetes and hypertension, there appears to be a consistent association between RAMs and CAD across diverse populations. This ultimately warrants further research to determine if RAMs are specific indicators of CAD and if they hold any independent predictive value. If these findings are validated in larger prospective cohorts with standardised imaging techniques, retinal imaging and the detection of retinal microaneurysms could become an integral part of cardiovascular risk stratification and preventive cardiology.
